# Supplementary material for: Are carbon emissions trading and green financial instruments synergistic? -Comprehensive quantitative research based on content analysis
Source: PLoS One. 2024 Mar 7;19(3):e0298601. doi: 10.1371/journal.pone.0298601 (PMC10919601; doi:10.1371/journal.pone.0298601)
Supplement: S1 Data — (ZIP) [file pone.0298601.s001.zip › Data/地级市Z_F工作报告.zip!/╜¡╦╒/╤∩╓▌╩╨/└┤╫╘╣1⁄2╓┌║┼í╛┬φ┐╦╩2╛▌═°í┐_2015.docx]

各位代表：

现在，我代表扬州市人民政府向大会作工作报告，请予审议，并请市政协各位委员和其他列席人员提出意见。

2014年工作回顾

2014年，我们以习近平总书记系列重要讲话精神为根本遵循，以人民群众过上幸福美好的生活为奋斗目标，在中共扬州市委的领导下，团结和依靠全市人民，全面落实省委八项工程、省政府十项举措，面对艰巨繁重的改革发展稳定任务，坚持改革激发活力和增强内生动力双驱动，做大增量和做优存量双促进，统筹协调和分类指导双结合，优化政策和狠抓落实双推动，保持定力，精准发力，较好地完成了市七届人大三次会议确定的目标任务。预计全年实现地区生产总值3625亿元，增长11%。公共财政预算收入295.19亿元，增长13.9%，增幅列全省第二；其中税收收入242.22亿元，增长13.9%。城镇常住居民人均可支配收入30380元，增长10%；农村常住居民人均可支配收入15255元，增长11%。“54321”重点工作当年任务圆满完成。节能减排实现省定目标。

1.深化改革取得新突破

抢抓政策机遇，全力推进跨江融合发展综合改革试点。实施跨江融合发展行动计划。深化与上海、苏南对接工作，全市开发园区与上海14家园区签署合作协议，落户亿元以上项目16个；18家苏南企业参与海峡两岸（扬州）农业合作试验区建设。《宁镇扬同城化发展规划》获省批准。完成宁扬城际轨道交通前期工作。争取省对宝应、高邮专项扶持政策，获得资金2亿元。突出简政放权，加快实施行政体制改革。清理并公布行政审批事项、行政权力、部门专项资金、行政事业性收费4项清单。改革食品药品管理体制，整合部门职责，执法重心下移。推进中介机构与行政机关、事业单位脱钩。对行业协会、商会等5类社会组织实行直接登记，试行注册资金认缴制备案登记。所有县（市、区）对外贸易经营者备案登记实现就地办理。围绕激发活力，不断深化经济体制、社会领域改革。推进国有资产监管全覆盖，完成市级行政事业单位所属国有企业清理整合工作。完成市属国有企业混合所有制改革项目41个。实行工商登记“先照后证”和放宽市场主体住所登记条件等多项举措。新增私营企业1.9万户、个体工商户3.9万户、民营企业注册资本金553亿元。完善事权与财权相匹配的市区财政管理体制，出台市区财政和生态补偿转移支付办法。完成事业单位分类改革。整合优化职教资源，成立职教集团。深化医药卫生体制改革，所有县级公立医院同步实施医药价格综合改革。初步建成市县乡三级农村产权交易平台。实现县乡村社会管理服务中心、五大专业调解组织和个人调解工作室“三个全覆盖”。

2.经济发展实现新提升

以重大项目为支撑，切实增加有效投入。全年完成固定资产投资2380亿元，增长19%。新开工重大工业项目51个。上海大众新增10万辆整车、尤妮佳二期、江淮10万辆皮卡等一批重大项目开工、投产。2012年以来竣工的44个重大工业项目实现开票销售、税收分别占全市工业的18%和19%。继续推进“122”技改工程，全年技改投入增长22%。新竣工重大服务业项目30个，新开工41个。以提质增效为目标，大力推进产业结构优化。规上工业增加值增长12%，全部工业开票销售、入库税收分别增长10%、12%。汽车、机械、化工、船舶、新能源新光源等五大千亿产业产值增幅全年均保持11%以上。建筑业实现产值2760亿元，增长12%。获鲁班奖1项。高邮获“中国建筑之乡”称号，邗建集团获房屋建筑工程施工总承包特级资质。服务业增加值占GDP比重提高1.5个百分点，达42.5%。旅游业实现总收入560亿元，增长17.7%。邗江瓜洲创成省级旅游度假区。软件和信息服务业完成业务收入420亿元，增长50%。新引进京东等5家全国百强电商企业。沿江港口完成货物吞吐量8500万吨、集装箱55万标箱。粮食产量实现“十一连增”。新建高标准农田24.26万亩。新增设施农（渔）业16.7万亩。新增规模经营15万亩。创成国家级示范合作社16个、省级示范家庭农场10个。县级以上农业龙头企业销售增长15.6%。以挖潜升级为重点，进一步扩大内外需市场。全年社会消费品零售总额1240亿元，增长12.8%。落实国家各类促进消费政策，推动汽车等大宗消费增长；住宿业、餐饮业零售额分别增长12%、12.5%。电子商务交易额230亿元，增长40%。房地产完成投资340亿元，商品房成交425万平方米。举办“烟花三月”国际经贸旅游节、世界运河名城博览会等活动，承办东北亚名人会、世界绿色设计论坛等10场国际会议。全年注册外资实际到账15亿美元，新增世界500强投资项目8个。完成外经营业额6.1亿美元。进出口总额100亿美元，其中出口76亿美元。

3.创新转型迈出新步伐

强化企业服务，优化发展环境。实施服务企业的2号文件及8个配套措施，51个事项实行容缺受理和模拟审批。实行工商执照、组织机构代码证、税务登记证“三证合一”登记制度。金融机构新增贷款390亿元，增长16.7%；企业直接融资163.4亿元，增长118%。通过设立科技型中小企业贷款风险补偿资金池、小微企业信贷引导资金池、政府应急专项资金池等举措，为企业发放各类贷款65亿元。实施“双百企业创新发展助推计划”，18家企业新列入省科技企业上市培育计划。成立北京产交所、上海联交所扬州工作站，为企业搭建资产、资本、技术交易平台。8家企业在“新三板”挂牌。顺利完成第三次经济普查。加快资源集聚，致力科技创新。成立以服务中小企业为宗旨、市场为导向、应用技术研发为重点的扬州产业技术研究院。“科教合作新长征”计划深入推进，签订校企产学研合作协议496项，新引进高校院所或知名企业研发中心、创新中心54家。清华扬州智能装备科技园、东大科技园、扬大科技园开园运行。新开工科技产业综合体22个，累计建成231万平方米、在孵企业521家。省级以上企业研发机构突破400家。创省级以上品牌79个、新产品638个。获全国“质量魅力城市”称号。牧羊集团获准筹建国际标准化组织饲料机械技术委员会。制（修）定国家或行业技术标准17项，专利授权1万件。市开发区获批国家级知识产权集群管理试点。全市新增高新技术企业92家，总数达524家。高新技术产业产值突破4000亿元，占规上工业总产值比重43.5%。引进高层次领军人才160名、专业技术人才1435名，引进培养企业高层次人才1516名。加强生态倒逼，推动绿色发展、集约发展。创成国家生态文明建设示范区。入围全国首批新能源示范市。出台《大气污染防治行动计划实施细则》。政府投入3亿元，淘汰小型燃煤锅炉162台（套）、淘汰报废老旧汽车18880辆，购置新能源公交车355辆，全部完成省下达的年度任务。开展建筑扬尘和烧烤油烟专项整治。实行工业集中区分类认定管理并启动生态工业园创建。加快发展节能环保产业。实施节能技术改造项目120项、循环经济项目20项，实现节能20万吨标煤。全市造林4.4万亩，市区新增绿地102.5万平方米。邗江、广陵创成省年度“国土资源节约集约模范区”。

4.城乡面貌呈现新变化

坚持交通先行，继续加大重大基础设施建设力度。连淮扬镇铁路扬州段率先开工，宁启铁路复线及电气化改造扬州段基本建成。宿扬高速公路、环邵伯湖大道开工建设，S203扬州段、S125仪征段建成通车，文昌路西延全线贯通。当年建成和在建干线公路总里程288公里、总投资23.6亿元，创历史新高。西部交通客运枢纽主体完工。高邮运东船闸、江都芒稻船闸扩建工程加快实施。淮河入江水道整治主体工程基本完成。完成古运河等中小河流整治。仪征套闸除险加固等工程完工。提升功能品质，不断增强中心城市首位度。100个重大城庆项目已建成38项，其中政府主导的30个项目全部达序时。瘦西湖隧道建成通车，新万福路、邗江路南延、金湾路有序推进。“双峰云栈”、大明寺西花园、鉴真广场等建成开放。实施8条城市劣V类河道整治，改造完成城市积水点18处。完成苏北医院、跃进桥、老北门桥等中环疏解节点道路改造工程。第一水厂提标扩建完工。建成清源污水处理厂二期。铺设污水管网30公里。赵庄垃圾填埋场增容和建筑垃圾综合利用处理场投入使用。推进“一流电网”建设，覆盖面积1491平方公里。推进“三拆三整治”，拆破拆烂拆违80万平方米。主城区数字化城管覆盖范围扩大到130平方公里。建设美好镇村，加快推进城乡一体化。9个乡镇入选全国重点镇，10个村创成新农村建设“优美乡村”。全市城镇化率61%。农村河道疏浚整治完成年度计划的116%，并全面实施河长制管护。更新改造区域供水支管网623.2公里。重点考核断面水质达到或优于Ⅲ类水质标准。新改建农村公路223公里、桥梁186座，镇村公交覆盖率65%。仪征、江都被评为省村级“四有一责”建设先进县（市、区）。76%的低收入农户实现脱贫。完成400个村土地承包经营权确权登记颁证工作。争取农业开发资金2.21亿元。粮食、农机、农业科研、供销、气象在“三农”工作中发挥重要作用。

5.社会建设取得新成效

聚焦民生幸福，大力实施社会保障和实事工程。新增城镇就业7.2万人、转移农村劳动力1.86万人，期末城镇登记失业率2.08%。城乡基本养老保险、基本医疗保险参保率均达98%。全面实施城镇居民大病保险制度，推进全市社会保险“一卡通”。连续10年提高全市企业退休人员基本养老金水平，人均每月已达到1955元。市区城乡低保标准统一提高至每月535元。设立市级急难家庭救助基金2000万元、困境儿童就学专项救助基金600万元。安置残疾人就业1581名，对791名残疾学生及贫困残疾人子女实施教育帮扶。新辟、优化公交线路21条，实行公交持IC卡1小时内免费换乘。邗江路公交专用道建成并试运行。市区公交分担率22.6%，比上年提高2个百分点。建成主城区公共自行车租赁系统，设立租赁服务点310个，投放车辆1万辆。整治“城中村”15块、老小区60万平方米，公房解危4.5万平方米。新增住房公积金缴纳人数7.8万人。新改建农贸市场15个。新建蔬菜生产基地2000亩，新增鲜奶4000余吨，新设农产品产地准出检测监控点20个。认定“三品”农产品120个。城乡居家养老服务覆盖率分别达100%、80%。注重共建共享，协调发展各项社会事业。大运河申遗取得成功，扬州10个遗产点和6段河道列入《世界遗产名录》。完成天宁寺文化核心区一期工程。新增3项国家级非物质文化遗产名录。首次承办中国工艺美术精品展和“中国电视演员形象榜”揭榜仪式。扬州曲艺赴京集中展演。扬剧《衣冠风流》获省“五个一工程”奖。组织文化惠民演出241场。集教学、研究、传承、展示等为一体的戏曲园开工建设。宝应、高邮、仪征、江都通过全国义务教育发展基本均衡县（市、区）国家级督导评估。新改建公办幼儿园6所。高考普通类本二上线率58.4%，比上年提高5个百分点。扬州技师学院被评为全国技能人才培养突出贡献单位。新农合筹资标准提高到人均400元，最高报销限额提高到18万元。在全省率先开展农村居民大病保险工作。实施“单独两孩”新政。市财政安排3400万元兑现2009年至2013年持独生子女证退休企业职工一次性奖励。宋夹城体育休闲公园全面建成开放。完成十八届省运会目标任务。扬州马拉松三度蝉联国际田联金标赛事。科普、档案方志、台湾事务、侨务、外事和工会、青年、妇女、儿童、老龄、慈善、红十字等事业取得新进步。强化社会治理，保障社会稳定。出台关于减轻社区负担、提升服务群众效能的六项规定，进一步厘清社区依法履职和协助工作事项。发放流动人口居住证4.5万张。7个社区矫正管理教育服务中心投入运行。我市被表彰为“六五”普法中期全国先进城市。强化涉稳评估和立体防控，社会治安持续稳定。全面完成青奥安保等重大任务。出台并实施古城消防安全管理办法。推进应急平台建设。建成并联网运行市、县隐患排查治理信息系统。组织开展危险化学品、建筑施工、特种设备等重点行业专项整治，实施“六打六治”打非治违专项行动，生产经营性事故数和死亡数分别下降4.14%、7.3%。出台军民融合发展意见并获得南京军区肯定。宝应“二妹子”民兵班扩编建连。民族、宗教、民防、地震、仲裁等工作取得新进展。

6.政府自身建设得到新加强

深入开展党的群众路线教育实践活动，切实改进工作作风。高标准严要求抓好学习教育、听取意见，查摆问题、开展批评，整改落实、建章立制等工作，着力解决关系群众切身利益的问题。制定出台加强依法科学民主决策、深化政务公开等10项制度。扎实推进拆迁安置房超期安置、区划调整中“同城同步同标”、行政审批“中梗阻”等突出问题整改措施的落实。开展减轻基层负担等专项整治行动。以市政府名义制发的文件和会议分别下降18%和17%，“三公”经费压减16.1%。打造阳光政府，深入推进政务公开。全面推进行政权力网上公开透明运行，在运行平台上共办理行政权力事项1980多万件。“净地出让、

摇号主持、现场直播、全程跟踪”的土地出让机制获省推广。“中国扬州”门户网站群服务范围拓展到15类主题218项事项。加强政府信息公开工作，及时公开“三公”经费信息和环境保护、安全生产、食品药品安全等公共监管信息。依法依规做好依申请公开工作。常态化、制度化做好政府新闻发布工作，全年召开新闻发布会178场。12345政府服务热线和“寄语市长”回复率96%、满意率88%以上。坚持依法行政，努力提升政府效能。自觉接受市人大及其常委会的法律监督、工作监督和市政协的民主监督，广泛听取各民主党派、工商联、无党派人士和社会各界意见。全年办理市人大代表议案、建议323件，政协提案432件，其中市长领办建议、提案13件。办理行政复议案件250件。制定市政府规范性文件9件。规范和完善重大行政决策程序。制定《“三直接”新十大环节操作规范》。组织开展“百企问政”和“三公开三报告”电视直播活动。对41个单体投资亿元以上国资项目驻点督查。

各位代表！以上成绩的取得，是中共扬州市委正确领导的结果；是市人大、市政协有效监督、大力支持的结果；是全市人民齐心协力、共同奋斗的结果。在此，我代表扬州市人民政府，向全市人民，向人大代表、政协委员，向各民主党派、工商联、无党派人士、各人民团体，向驻扬部队、驻扬单位，向所有关心、支持、参与扬州建设和发展的同志们、朋友们，表示崇高的敬意和衷心的感谢！同时，我们清醒地看到经济社会发展中还存在一些矛盾和问题，主要是：稳增长调结构促转型需要付出艰苦努力；内需不旺和经济外向度不高并存；区域协调发展与城乡统筹发展仍有大量工作要做；民生改善和公共服务水平与百姓期盼还有差距；生态文明建设与可持续发展的要求不相适应；法治政府和政府作风建设、反腐倡廉任务依然艰巨。对此，我们必须高度重视，并采取切实措施认真加以解决。

2015年工作安排

今年是全面深化改革的关键之年，是全面推进依法治市的开局之年，是“十二五”规划的收官之年，也是扬州建城2500周年的喜庆之年。

我们要认真学习贯彻习总书记对江苏工作提出的“经济强、百姓富、环境美、社会文明程度高”的最新要求，振奋精神，积极作为，切实增强工作的创新力、执行力、协同力，牢牢把握转型发展、创新发展的主动权。

今年政府工作总体要求是：全面贯彻落实党的十八大、十八届三中、四中全会、中央经济工作会议精神和习近平总书记系列重要讲话精神，按照省委十二届八次、九次全会和市委六届八次全会的决策部署，主动把握和积极适应新常态，坚持“稳中求进、进字当先，创新驱动、转型升级”工作总基调，全面深化改革，全面推进依法治市，着力深化产业结构调整，着力强化生态文明建设，着力提升城市建设和管理水平，着力保障和改善民生，着力筑牢风险防控底线，努力保持经济社会平稳健康发展，加快建设古代文化与现代文明交相辉映的名城。2015年全市经济社会发展的主要预期目标为：地区生产总值增长10%，公共财政预算收入增长10%，社会消费品零售总额增长12%，固定资产投资增长17%，全社会研发投入占地区生产总值比重2.5%左右，居民消费价格指数低于全省平均水平，城镇、农村居民人均可支配收入均增长10%，城镇登记失业率控制在3%以内，节能减排完成省下达任务。

重点抓好以下八个方面工作：

1.坚持项目支撑和创新引领，加快推动产业结构优化升级

围绕提升经济发展质量和效益，落实市委“产业项目升级年”的要求，全力推动增长动力转换和经济结构再平衡。

促进重大项目提质增效。紧盯跨国公司、央企和知名民企，主攻基地型、科技型、区域总部型项目和重大技改项目，加快实现沿江100亿、沿河50亿投资项目第二轮全覆盖。全面实施重大项目质效评估。围绕项目技术水平、装备和研发投入、资源能源消耗和环境影响、产出效益和社会贡献等重点优化项目落地。全年新开工10亿元或1亿美元重大工业项目50个、重大服务业项目40个，基本产业和“双高”项目占比均达60%以上。全力推进科技创新战略。深化国家创新型城市建设。强化科技同经济对接。新增国家高新技术企业60家，高新技术产业产值占规上工业总产值比重44%以上。新设科技金融专营机构3家、创投基金5支。强化创新成果同产业对接。全面提升产业技术研究院的带动作用。建立区域技术交易市场，举办“中国（扬州）重大科技成果展示洽谈会”。开发具有自主知识产权的高新技术产品500项以上。科技综合体入驻企业700家，使用率达70%。强化创新项目同现实生产力对接。实施“科技产业合作远征计划”，深化与扬大等高校在科技、人才等方面的对接合作，企业与高校院所签订合作协议400项，新建省级以上“三站三中心”30家。继续推动知识产权质押融资试点，新增知识产权贯标企业30家。强化创新人才培养引进同经济社会发展需求相对接。落实“6+1”人才引进和培育政策，用好用活各类人才，新引进高层次领军人才100名、产业发展急需专业技术人才1000名。推动制造业向高端攀升。加快汽车核心技术和配套件集聚与提升，推动新能源汽车关键技术研发及产业化应用。实施50家机械装备企业向机器人产业转型等“五个五”工程，加快信息技术在智能装备制造中的集成和应用，大力发展工业机器人产业。促进船舶产业存量优化提升，培育高技术特种船舶和海洋工程装备制造业。推进海峡两岸（扬州）绿色石化产业合作区建设。引导新能源新光源骨干企业优化产品结构。大力发展食品工业，强化龙头企业引进与培育，加快品牌打造和市场开拓，推进食品工业园发展生产研发、工业旅游、冷链物流等新型业态。规上工业增加值、入库税收分别增长11%、9%。促进服务业创新突破。出台互联网产业发展三年行动计划。加快基于互联网产业的软件自主研发企业以及企业线上销售平台建设，业务收入增长50%。强化现代金融业发展模式创新，新引进股份制银行1-2家，支持现代金融集团、扬州农商行发展。坚持“城市即旅游”，强化市县联动、项目带动，引进1-2个重大旅游项目，推进7个重点旅游度假区建设，旅游业增加值占GDP比重7.5%左右。推动文化产业与科技、创意、金融、旅游、体育等融合发展，着力打造工艺坊等文化产业集聚区，培育一批文化科技企业，在文化载体、产品、市场上有新突破。注重医养结合，吸引各类资本投入，大力发展健康养老产业。通过引导刚性需求、发展高性价比住房、促进租售市场建设等措施，全力保持房地产市场平稳健康发展。加快现代物流业发展，推动空港新城及航空物流、扬州港“亿吨大港、百万标箱”建设。服务业增加值占GDP比重43%以上。

2.继续深化改革和扩大开放，激活发展内生动力

正确处理政府有形之手与市场无形之手关系，用好内外两个市场、两种资源，着力打造转型发展新引擎。

继续深化重点领域改革。扎实抓好已实施的重点改革事项落地见效。完成旅游和文博资源管理利用体制改革。坚持以“管资本”为重点加强国资监管，探索推进国有资产证券化，建立健全现代企业制度和国有资本经营预算制度。大力推动与政府相关的行业中介、社团组织、经营实体市场化改革。推进公共资源交易中心实体化运作。按照“同级同权同责”原则，构建好新型市区财政关系。尊重基层首创，及时发现总结各地创新实践。高度重视、积极防范政府性债务、金融、房地产等领域的潜在风险，牢牢守住不发生区域性系统性风险的底线。着力优化投资消费结构。加大对高端装备制造、生产性服务业、节能环保以及农田水利的投入，完成全社会固定资产投资2600亿元以上。进一步提升批零住餐等传统消费，以模式创新做大冶春、谢馥春等老字号。明确新建商业综合体的特色定位。稳定住房、汽车等大宗消费，扩大文化、旅游、信息、健身养生等新兴消费。加快电商平台建设，大力推进线上消费，探索O2O新型商业模式，全年电子商务交易额300亿元。启动创建国家电子商务示范城市。积极畅通商品流通环节，打造更加安全、舒适、便捷的消费环境。努力提高经济国际化水平。围绕企业、城市、人才“三个国际化”，提高参与国际经济合作、竞争的能力。坚持“引进来”与“走出去”并举，继续实施“530”计划，大力推进产业链和企业招商，新落户世界500强投资项目5个以上，注册外资实际到账15亿美元。推动一批重点外经企业入围省本土型跨国企业培育名录，外经营业额增长15%。提升电子口岸和通关“三个一”建设水平，外贸出口80亿美元。实现扬泰机场一类口岸开放运营并尽快开通国际航班。积极承办重要国际性会议会展和商务活动。主动参与“一带一路”和长江经济带建设。

3.做优做强市场主体和功能载体，切实增强发展后劲把稳企业、稳实体作为稳增长的基础，特别是服务好小微企业，扎实抓好发展难题破解，努力培植经济新增长点。持续增强企业核心竞争力。继续制定实施服务企业的2号文件。强化“人才、科技、资本”联动，加大对企业科技研发人员引进和培育力度，实现企业研发经费占主营业务收入比重、企业研发人员比重“双提升”。深入推进“千企技改”和百家重点企业技改提升“532”工程。实施科技企业培育“168”工程、创新型企业家培育“青蓝计划”。强化企业“标准、品牌、专利”工作，制（修）定国家、行业和地方标准18项以上，创成省级以上品牌45件，争取授权专利1万件。争创“全国质量强市示范城市”。推进小微企业公共服务平台建设，充分发挥现有中小企业专项资金的引导作用，积极引导创业投资基金、天使基金、种子基金投资小微企业，鼓励中小企业“专、精、特、新”发展，打造一批行业“小巨人”。充分释放民营经济发展活力。持续推进工商登记制度改革，扩大“先照后证”项目范围，建立统一的市场监管体制。发展混合所有制经济，营造鼓励民资发展的政策环境，推进政府主导的项目向民资开放。积极推动宜创城市建设，切实降低门槛、强化保障，鼓励和吸引更多创业者在扬州就业、置业、创业。全年新发展私营企业1.8万户、个体工商户3万户，新增民营企业注册资本金实际到资550亿元、开工亿元以上项目120个。

推动园区转型升级、创新发展。把市经济开发区打造成转型升级、创新发展的先导区和示范区，切实提高贡献份额。加快推动扬州高新区争创国家级高新区、江都经济开发区争创国家级开发区，生态科技新城和高邮湖西新区争创省级高新区。各省级以上开发区与上海、苏南园区开展有集群效应、集聚成果的实质性合作，分别承接过亿元转移项目3个以上。推动中外合作园区建设，把广陵“中德液压产业园”、市开发区“中瑞（典）生态产业园”办成国内外有影响的园区。

4.加快农业现代化，推进新型城镇化和城乡一体化

把“三农”工作放到更加突出的位置，坚定不移加快农业发展方式转变，在城乡互补、工农互惠的道路上迈出新步伐。

大力发展现代农业。加快构建农业一二三产融合发展体系，切实提高农业技术装备和设施化水平。实施粮食高产增效工程。加快粮食现代物流体系建设。持续推进6个50亿元连片特色农业产业基地建设，新增高标准农田15万亩、设施农业8万亩、设施渔业4万亩。放大驻扬农业科研院校技术优势，推动园艺、畜禽等特色产业发展。规划建设扬州现代农业科技园。把产业链、价值链等现代产业组织方式引入农业，推动农副产品深加工，扶持农业龙头企业做大做强。加快农产品直销合作社、平价店、直通车等流通载体建设。农产品加工业总产值增长20%。加强农产品安全监测，地产农产品质量抽检合格率98%以上。深化农村改革。300个村完成农村土地承包经营权确权登记颁证。规范有序推进土地流转，新增土地集中型农业适度规模经营10万亩。探索土地入股、托管等多种方式，大力培植新型农业经营主体，新增20个市级示范家庭农场，重点培育100个示范合作社。推进供销社综合改革。完善农村产权交易市场体系建设。深化农村集体资产股份合作制改革，培育规范运作、制度完善、与农民结成利益连接体的村经济合作社（社区股份合作社）40个。稳步推进农村金融改革，加快开展“三权”抵押贷款。提升农业保险服务水平，主要种植业参保面90%以上。优化城镇乡村建设。坚持“三规合一”“四化同步”，编制新型城镇化及城乡一体化规划。分类推进卫星镇、特色镇、一般镇建设，每个县（市、区）集中力量规划建设1-2个10万人以上规模的现代新城镇。鼓励和吸引社会资本投入城镇化建设。高度重视农村生态环境的治理和保护，深化美丽乡村建设，创成“优美乡村”10个，完成60个村环境连片整治。新增改建农村卫生户厕4万座。新改建农村公路185公里，改造农村危桥85座，镇村公交开通率达72%。推动城市公共服务向镇村延伸。有序推进农村转移人口市民化，力争城镇化率提高1个百分点。做大做强县域经济。进一步落实好省支持宝应、高邮的专项政策。推进仪征宁扬城际轨道项目开工以及后山区的生态保护和有序开发。加快环邵伯湖大道、S352江都段、S331宝应段、S333高邮段、界首运河大桥等重点基础设施建设。强化产业特色发展、集群发展，加快实现县（市）争创省级高新区（科技园）全覆盖。提升工业集中区生态化、集约化、特色化发展水平。已认定的工业集中区开票销售增幅比上年提高6个百分点，其中特色产业园占比35%、生态工业集中区占比20%。新建各类公共服务平台10家。沿江、沿河地区80%的村集体经营性收入分别达到50万元、40万元。严格控制并着力化解镇村债务。

5.切实加强环境保护，全面提升生态文明建设水平

全力推进“生态文明建设突破年”各项工作，加快国家级生态文明建设试点示范市和水生态文明建设示范市创建，让生态成为市民的永续福利，生态文明建设走在全省前列。加大“治水”“治气”力度。城区主干河道活水和全市域农村河道疏浚实现全覆盖，并强化河长制管理。完成扬州闸、黄金坝闸、平山堂泵站等建设及市区8条劣V类河道综合整治。加快推进主城区污水管网“四统一”，新铺设管网28.7公里。六圩污水处理厂三期厂区建成。严格落实省大气污染防治十项行动。淘汰替换小型燃煤锅炉209台，提标改造火电、水泥等行业企业44家。完成黄标车淘汰任务。严格强化渣土运输管控。古城区、瘦西湖景区核心区和居民小区内全面取缔露天烧烤。出台秸秆收贮和利用的扶持政策，秸秆综合利用率92%以上。PM2.5年平均浓度在2013年基础上下降7%。推动生产生活方式绿色转型。实施重点减排工程110项，完成二电厂1号、3号燃煤机组超低排放改造。加快推进扬农化工、联环药业、威亨热电、裕华织造等企业转型搬迁。确保完成“十二五”期间单位GDP能耗下降17%目标。进一步提升环保科技产业园发展水平。化工园区严格“四至”范围，提高准入门槛，争创省级生态工业园区。落实节约集约利用土地二十条措施，盘活闲置、低效利用土地8000亩。开展绿色建筑行动，全市城镇新建建筑全面执行省绿色建筑设计标准。鼓励和扶持建筑企业晋升资质、质量创优，全年建筑业产值增长12%。完善绿色交通体系，新增新能源汽车800辆。加强生态涵养。实施新一轮“绿杨城郭新扬州”三年行动计划。规划建设纵贯扬州南北的江淮生态大走廊，扎实做好南水北调水源地、沿江沿运河等区域的环境保护以及城郊结合部的环境整治，加快建设廖家沟中央生态公园、三湾城市生态公园和县（市、区）10平方公里生态中心。恢复湿地4000亩。严格落实市人大常委会决议，加快“七河八岛”区域沿河船厂砂石场搬迁。继续提升“五路一环一河”绿化品质。市区新增城市绿地100万平方米，全市新增立体绿化11万平方米。实施村庄绿化、农田防护林、高速公路出入口提升等五大林业重点工程，新建省级绿化示范村40个，全市新增成片林2万亩。严控农业面源污染。完善政策体系。按照《扬州市生态文明建设规划》，制定生态文明建设三年行动计划。引导社会资本参与生态建设。市财政专项用于生态建设投入10亿元以上。研究出台鼓励绿色出行、低碳消费、循环利用等办法。建立系统的绿色发展水平评估体系。实施生态红线区域生态补偿政策。探索排污权有偿使用和交易机制。加大生态指标考核权重，树立鲜明的绿色发展导向。严格执行新《环保法》，提高环保执法刚性要求。

6.围绕建城2500周年，进一步提升城市品质和文化影响力

按照市委“城建项目决胜年”、“城市管理强化年”和“市民素质提升年”部署，推动城市硬件设施和软实力双提升、人与城市共发展。

加快重大基础设施建设。宁启铁路复线电气化改造扬州段全线建成，全面启动连淮扬镇高铁枢纽站及其综合体规划建设。加快推进宿扬高速公路建设和京沪高速公路扩容及南延过江前期工作，进一步完善“一环七射”高速公路网。加快构建城市骨干路网体系，新万福路、沙湾路南延、S353扬州西段等建成通车，加快推进邗江路南延、金湾路、开发路东延等道路建设。建成投运500千伏扬州北输变电工程。全力提升城市建设和管理水平。突出建管用一体，继续推动100项城庆重大项目建设，着力把政府主导的30项城建项目打造成精品和示范。围绕“宜居、宜业、宜创”，突出生态、低碳、集约，进一步提升生态科技新城和广陵新城建设发展水平。全面建成城庆广场、市民中心等功能性设施和交通银行金融服务中心、环球金融城等产业综合体。规划实施蜀冈中西峰自然生态修复、历史风貌恢复工程，加快蜀冈-瘦西湖景区环境提升。切实改善古城居民生活条件。坚持专项整治与长效治理相结合，集中开展流动摊点、乱行乱停等十项整治行动，完成省城市环境综合整治三年行动目标任务。县（市、区）全面建成数字化城管。加大环卫基础设施投入，建成餐厨垃圾处理场、生活垃圾焚烧发电二期工程，改建垃圾中转站3座。加强城市道路堵点改造，提升交通通畅性。

进一步彰显名城文化魅力。放大大运河世界遗产效应，规划建设大运河文化展示园、阮元广场等一批主题文化广场，加快实施隋炀帝墓考古遗址公园等重点工程。突出现有文博场馆管理和利用，提升文化博览城建设水平。完成全国第一次移动文物普查。采取更加有力措施集聚非物质文化专门人才。编纂出版《扬州文库》等地域文化丛书。推进“书香城市”建设。实施城庆精品创作等工程。设立扬州市文学艺术奖。继续办好“烟花三月”国际经贸旅游节、鉴真国际半程马拉松赛、世界运河名城博览会等品牌活动和国际性会议，举办建城2500周年系列城庆活动。加强社会主义核心价值观教育，大力选树、学习宣传当代“扬州好人”和先进模范。

7.突出稳定性、连续性和累积性，继续推进民生幸福工程

坚持守住底线、突出重点、完善制度，像抓经济建设一样抓民生工作，加快推动民生工作再上新台阶。

千方百计增加居民收入。以劳动者素质提升带动收入提高，实现更加充分、更高质量就业。城乡劳动者职业技能培训2.25万人、创业培训4000人，新增高技能人才1.42万人。全年新增城镇就业5万人、农村劳动力转移1.4万人。扬州籍高校毕业生就业推荐率和有就业意愿的困难家庭毕业生就业率均达100%。新建创业孵化基地10个。多措并举提高居民工资性、经营性、资产性、保障性收入。探索古城保护与市民创业增收相结合的新路径。实现建档低收入农户人均纯收入5000元的脱贫目标。

稳步提高社会保障水平。城乡基本养老保险、城镇基本医疗、工伤、生育保险参保分别新增3万人、2万人、2万人和3万人。新农合参合率稳定在97%以上，政策范围内住院补偿比达75%。完善大病保险制度和医疗救助保障机制，深化医保支付方式改革。继续推进市区社保“同城同步同标”，市区新农合实现“五个统一”。进一步完善安全有效、方便廉价的公共卫生和基本医疗服务体系。坚持社区卫生服务与居家养老相结合，提升已建居家养老服务中心（站）利用率和服务水平。完善困境儿童助学帮扶体系。实行城乡低保、农村五保供养标准自然增长机制。进一步做好残疾人保障和服务工作。

扎实做好重点民生工作。完善多层次住房供应体系。市区实施公有住房解危4.5万平方米，新建（筹集）公租房1400套，完成征收（拆迁）超腾仓期未安置住房任务。完成老小区综合整治55万平方米，整治“城中村”15个。推进物业服务标准化、物业监管信息化，老旧小区新增基本物业服务68万平方米。新改建农贸市场10个。改造博物馆路等8个积水点。新辟、优化调整公交线路16条，公交分担率达26%。加密公共自行车租赁点97个。建设荷花池等停车场。继续推进“1161”菜篮子工程和“115”鲜奶工程。

加快发展社会事业。推进教育公平与内涵建设。新（改）建公办幼儿园6所，新创省义务教育现代化学校60所，热点高中70%招生名额面向区域内初中学校分配。建成市特殊教育学校新校区。启动市级公立医院综合改革。加快推进分级诊疗制度。推广苏北医院优质医疗资源与基层医疗单位结对的做法，逐步缓解大医院人满为患状况。启动实施农村区域医疗卫生中心建设。孕前优生健康检查覆盖率90%以上。举办市第十二届运动会，申办省第十九届运动会并规划建设全市域公共体育设施。实施三年农村健身设施提档升级工程，推进标准化体育休闲公园县（市、区）、功能区全覆盖。

8.进一步改善和创新社会治理，保持社会和谐稳定

积极适应百姓的新要求、新期待，全面提升社会治理科学化水平，为建设“人民满意、社会公认”的法治名城奠定基础。夯实基层基础工作。继续深化城乡和谐社区建设。按照“两清单、一协议、双评估”要求，全面推进“政社互动”。建成社区综合信息管理服务平台。以政府购买服务为主要方式，提高公共服务的覆盖面和质量水平。全面开展社会组织等级评估。完善社会工作人才队伍培养、使用和激励机制。全面实施社会诚信体系建设，完善扬州社会信用信息综合征信系统，增强企业信用体系追溯效能。维护群众合法权益。完善群众诉求表达和权益维护机制，整合拓宽民意表达渠道，引导和支持群众理性表达诉求、依法维护权益。完善法律援助制度，建成覆盖城乡的公共法律服务体系。推进普遍建立法律顾问制度和公职律师工作。实施诉访分离，建立律师参与涉法涉诉信访化解新机制。着力解决突出信访问题，包案负责逐一化解信访积案，落实涉法涉诉信访依法终结制度。加强医患纠纷、道路交通事故等重点领域专业性人民调解组织建设，推动人民调解与司法调解、行政调解、仲裁对接联动，努力将矛盾化解在萌芽状态。保持社会和谐稳定。继续实施“六五”普法规划。加强社会治安综合治理，完善社会治安防控体系，加强基层政法队伍建设和保障投入，提升平安扬州建设水平。完善市级社区矫正指挥中心功能。落实安全生产责任制，重大事故隐患挂牌督办和按期整改率100%，事故起数和死亡人数继续保持“双下降”。强化食品药品安全源头监管、风险防控和专项整治。推进食品药品安全电子监管工作。健全应急管理机制，提高突发公共事件的预警和处置能力。加强防灾减灾体系建设，提高气象、地震等灾害防御能力。进一步加强国防动员和后备力量建设，促进军民融合深度发展，争创全国双拥模范城“七连冠”。

全面推进法治政府建设贯彻落实十八届四中全会精神，强化法治思维、运用法治方式推动改革发展稳定各项工作，加快建设职能科学、权责法定、执法严明、公开公正、廉洁高效、守法诚信的法治政府。

1.全面依法履职，切实增强政府工作公信力和执行力

严格执行市人大常委会关于讨论、决定重大事项的规定。推行行政审批事项等“四个清单”制度，清理并公布部门责任清单，推进行政机构、职能、权限、程序、责任法定。以提高政府抓经济工作的法治化水平来适应经济新常态，争创发展新优势。坚持公平公正，以政府诚信带动社会诚信建设。完成市区管理体制“同级同权同责”。强化求真务实和监督问责，对确定的目标、制定的政策、作出的承诺，确保说到做到、干成干好。

2.突出公开透明，不断强化对行政权力的制约和监督

主动接受市人大法律监督、工作监督和市政协民主监督，加强与市政协的民主协商，认真听取各民主党派、工商联、无党派人士和社会各界意见。切实做好人大代表议案、建议和政协提案办理工作。扎实抓好财政资金使用、公共资源配置、公共服务、公共监管信息的公开。继续加强政府新闻发布工作。办好12345政府服务热线、“寄语市长”栏目。推进行政复议委员会试点。继续强化行政权力网上公开透明运行，做到省市县三级权力事项“四个统一”。健全司法监督、审计监督、舆论监督制度，增强监督合力和实效。

3.强化依法行政，加快健全依法决策机制和执法体制

认真落实公众参与、专家论证、风险评估、合法性审查、集体讨论决定的重大行政决策法定程序，并全程留痕。积极做好政府行政立法准备。推动参与式行政程序建设。建立政府法律顾问运行机制。深化行政执法体制改革，推进食品药品、工商质监、公共卫生、安全生产等领域综合执法。探索功能区区域综合执法试点。严格实行行政执法人员持证上岗和资格管理制度。推广“五位一体”联合巡查、综合执法模式。完善市县两级行政执法管理。落实新《预算法》各项要求，通过强化预算约束倒逼行政行为的规范。

4.坚持勤政廉政，努力提高行政效能和服务水平严格落实中央“八项规定”，持之以恒纠正和解决“四风”方面存在的突出问题。厉行节约、勤俭办事，运转性支出在上年基础上统一削减5%、“三公”经费下降10%。全面推进公务用车改革。强化从源头预防腐败，继续制定《“三直接”新十大环节操作规范》，做到关键环节规范全覆盖。不断增强政府工作人员主动作为、敢于担当的意识。深化“三减一提”行政效能提升行动。全面试行重大项目联合会办、联合勘查、联合验收机制。坚持问需于民、问计于民，进一步优化服务流程，提高服务实效。加强战略谋划和务实论证，编制出一份更加适应时代要求、更加符合扬州实际、更加反映人民意愿的“十三五”规划。

各位代表！圆满完成今年和“十二五”规划的各项任务，共同谋划“十三五”的美好蓝图，我们使命崇高，责任重大。让我们紧密团结在以习近平同志为总书记的党中央周围，在省委、省政府和中共扬州市委的正确领导下，解放思想、锐意进取、真抓实干，让我们的城市一年比一年更美好，人民一年比一年更幸福，为谱写好中国梦的扬州新篇章而努力奋斗！
